# Correction: Metabolic activity of CYP2C19 and CYP2D6 on antidepressant response from 13 clinical studies using genotype imputation: a meta-analysis

**DOI:** 10.1038/s41398-024-03064-x

**Published:** 2024-08-31

**Authors:** Danyang Li, Oliver Pain, Chiara Fabbri, Win Lee Edwin Wong, Chris Wai Hang Lo, Stephan Ripke, Annamaria Cattaneo, Daniel Souery, Mojca Z. Dernovsek, Neven Henigsberg, Joanna Hauser, Glyn Lewis, Ole Mors, Nader Perroud, Marcella Rietschel, Rudolf Uher, Wolfgang Maier, Bernhard T. Baune, Joanna M. Biernacka, Guido Bondolfi, Katharina Domschke, Masaki Kato, Yu-Li Liu, Alessandro Serretti, Shih-Jen Tsai, Richard Weinshilboum, the GSRD Consortium, the Major Depressive Disorder Working Group of the Psychiatric Genomics Consortium, Andrew M. McIntosh, Cathryn M. Lewis

**Affiliations:** 1 Social, Genetic and Developmental Psychiatry Centre, King’s College London, London, GB UK; 2 Cancer Centre, The First Affiliated Hospital of Xi’an Jiaotong University, Xi’an, CN China; 3 Maurice Wohl Clinical Neuroscience Institute, Department of Basic and Clinical Neuroscience, Institute of Psychiatry, Psychology and Neuroscience, King’s College London, London, GB UK; 4 Department of Biomedical and Neuromotor Sciences, University of Bologna, Bologna, Italy; 5 Department of Pharmacology, Yong Loo Lin School of Medicine, National University of Singapore, Singapore, Singapore; 6 Department of Psychiatry and Psychotherapy, Universitätsmedizin Berlin Campus Charité Mitte, Berlin, DE Germany; 7 Analytic and Translational Genetics Unit, Massachusetts General Hospital, Boston, MA USA; 8 Biological Psychiatry Laboratory, IRCCS Fatebenefratelli, Brescia, Italy; 9 Department of Pharmacological and Biomedical Sciences, University of Milan, Milan, Italy; 10 Laboratoire de Psychologie Medicale, Universitè Libre de Bruxelles and Psy Pluriel, Centre Européen de Psychologie Medicale, Brussels, Italy; 11 University Psychiatric Clinic, University of Ljubliana, Ljubljana, Slovenia; 12 Department of Psychiatry, Croatian Institute for Brain Research, University of Zagreb Medical School, Zagreb, HR Croatia; 13 Psychiatric Genetic Unit, Poznan University of Medical Sciences, Poznan, Poland; 14 Division of Psychiatry, University College London, London, GB UK; 15 Psychosis Research Unit, Aarhus University Hospital - Psychiatry, Aarhus, Denmark; 16 Department of Psychiatry, Geneva University Hospitals, Geneva, CH Switzerland; 17 Department of Genetic Epidemiology in Psychiatry, Medical Faculty Mannheim, University of Heidelberg, Central Institute of Mental Health, Mannheim, Denmark; 18 Department of Psychiatry, Dalhousie University, Halifax, NS Canada; 19 Department of Psychiatry and Psychotherapy, University of Bonn, Bonn, Denmark; 20 Department of Psychiatry, University of Münster, Münster, Denmark; 21 Florey Institute for Neuroscience and Mental Health, University of Melbourne, Melbourne, Australia; 22 Department of Psychiatry, Melbourne Medical School, University of Melbourne, Melbourne, Australia; 23 Department of Psychiatry and Psychology, Mayo Clinic, Rochester, MN USA; 24 Department of Quantitative Health Sciences, Mayo Clinic, Rochester, MN USA; 25 Department of Psychiatry and Psychotherapy, Medical Center, University of Freiburg, Freiburg, Denmark; 26 Department of Neuropsychiatry, Kansai Medical University, Osaka, Japan; 27 Center for Neuropsychiatric Research, National Health Research Institutes, Miaoli, Taiwan; 28 Department of Medicine and Surgery, Kore University of Enna, Enna, Italy; 29 Department of Psychiatry, Taipei Veterans General Hospital, Taipei, Taiwan; 30 Division of Psychiatry, School of Medicine, National Yang-Ming University, Taipei, Taiwan; 31 Department of Molecular Pharmacology and Experimental Therapeutics, Mayo Clinic, Rochester, MN USA; 32 Division of Psychiatry, University of Edinburgh, Edinburgh, GB UK; 33 Department of Medical & Molecular Genetics, King’s College London, London, GB UK

**Keywords:** Depression, Predictive markers

Correction to: *Translational Psychiatry* 10.1038/s41398-024-02981-1, published online 19 July 2024

In this article the author’s name Chiara Fabbri was incorrectly written as Fabbri Chiara. The original article has been corrected.

